# Transforming the On-Call Psychiatric Handover: A Quality Improvement Project Using Microsoft Teams to Optimise Ease of Use, Clarity and Efficiency

**DOI:** 10.1192/bjo.2026.11422

**Published:** 2026-06-30

**Authors:** Nancy Liu, Robin Holliday, David Headden

**Affiliations:** 1 Dykebar Hospital, Glasgow, United Kingdom; 2 Leverndale Hospital, Glasgow, United Kingdom; 3 Glasgow Royal Infirmary, Glasgow, United Kingdom

## Abstract

**Aims:**

Effective handovers are crucial for patient safety and continuity of care in psychiatry. The Excel-based handover format used across three South Glasgow-linked hospitals (Dykebar, Leverndale and Inverclyde) frequently contained unresolved or outdated tasks, and did not clearly indicate urgency or ownership. Previous audits highlighted inefficiencies in this system, revealing an opportunity for innovation. We aimed to introduce a new Microsoft Teams handover, to improve clarity and efficiency and support safe on-call working across all three sites.

**Methods:**

Between December 2024 and March 2025, feedback was collected from resident doctors across three South Glasgow psychiatric hospitals via anonymised Likert scale and free-text surveys, to explore their experiences with the Excel handover and openness towards trialling a new Teams format. The handover was created using Planner software, utilising tabsand columns to organise wards. Advice was sought from Information Governance regarding use of patient data, and subsequently implemented: the system was made private with closely managed access, and a retention period was applied via automated workflows to transfer completed tasks onto a private Excel sheet every three months, to be stored locally for audit trail. Clinical directors and residents received demonstrations and tutorials, and approved of the launch. The Teams system was implemented across all three sites in April 2025. Post-intervention feedback was collected in May 2025 to gauge opinions on the new handover; following this, the system remained in use and was later replicated at other Glasgow hospitals with our guidance on setup and workflow.

**Results:**

All eight participants in the preliminary survey indicated they found the Excel handover had become inefficient and unclear, and were interested in trialling the new system. Following launch of the Teams handover, all 15 respondents agreed it brought improved usability, clarity, and clinical efficiency. Comments acknowledged the clean interface and the ability to check tasks off, assign due-dates, and schedule repeating tasks ahead of time. Later in 2025, resident doctors in North Glasgow reached out and successfully adapted this project at Gartnavel and Stobhill hospitals, demonstrating positive change across multiple areas of the health board.

**Conclusion:**

The findings provide evidence of improved satisfaction with the on-call handover since our introduction of the Teams interface. It is suggested that resident doctors value clarity and efficiency for smooth, safe team-working. The small sample size and single-region implementation limit generalisability, but feedback shows the project has brought a positive change to clinical work and has now been implemented at multiple hospitals.

